# Characteristics and Correlates of Recent Successful Cessation Among Adult Cigarette Smokers, United States, 2018

**DOI:** 10.5888/pcd17.200173

**Published:** 2020-12-10

**Authors:** Kimp Walton, Teresa W. Wang, Yvonne Prutzman, Ahmed Jamal, Stephen D. Babb

**Affiliations:** 1 Office on Smoking and Health, National Center for Chronic Disease Prevention and Health Promotion, Centers for Disease Control and Prevention, Atlanta, Georgia; 2 Division of Cancer Control and Population Sciences, National Cancer Institute, Bethesda, Maryland

## Abstract

We assessed characteristics and correlates of recent successful cessation (quitting smoking for 6 months or longer within the past year) among US adult cigarette smokers aged 18 years or older. Estimates came from the July 2018 fielding of the 2018–2019 Tobacco Use Supplement to the Current Population Survey (N = 26,759). In 2018, 7.1% of adult smokers reported recent successful cessation. Recent successful cessation varied by certain demographic characteristics, noncigarette tobacco product use, smoke-free home rules, and receipt of advice to quit from a medical doctor. To help more smokers quit, public health practitioners can ensure that evidence-based tobacco control interventions, including barrier-free access to evidence-based cessation treatments, are reaching all tobacco users, especially those who face greater barriers to quitting.

**Summary**

**What is already known on this topic?**

Increasing smoking cessation reduces smoking-related disease, death, and economic costs.

**What is added by this report?**

In 2018, 7.1% of US adult smokers reported recent successful quitting. However, some groups had less success, including certain demographic groups, and some groups had greater success, including exclusive e-cigarette users, people with smoke-free home rules, and people who received advice to quit from a medical doctor.

**What are the implications for public health practice?**

To help more smokers quit, public health practitioners can ensure that evidence-based tobacco control interventions, including barrier-free access to evidence-based cessation treatments, are reaching all tobacco users, especially those who face greater barriers to quitting.

## Objective

Increasing smoking cessation reduces smoking-related disease, death, and economic costs (1,2). Understanding the factors that contribute to successful cessation can inform public health strategies to help smokers quit successfully (2). By using nationally representative data from the Tobacco Use Supplement to the Current Population Survey (TUS-CPS), we assessed characteristics and correlates of adult cigarette smokers who quit smoking for 6 months or longer in the past year.

## Methods

Data came from the July 2018 fielding (the first of 3 data collections) of the 2018–2019 TUS-CPS, a cross-sectional, household-based survey of noninstitutionalized US adults aged 18 years or older in the 50 US states and the District of Columbia (3). TUS is administered every 3 to 4 years as part of CPS, a monthly survey conducted by the US Census Bureau for the US Bureau of Labor Statistics (4). In July 2018, 26,759 adults completed the TUS-CPS interview as self-respondents (mean self-response rate, 57.7%).

Current smokers were defined as adults who had smoked at least 100 cigarettes during their lifetime and currently smoked “every day” or “some days.” Former smokers were adults who had smoked at least 100 cigarettes during their lifetime but currently did not smoke at all. Recent successful smoking cessation was defined as former smokers who quit smoking cigarettes within the past year and remained quit for 6 months or more. Recent successful cessation was assessed among former smokers who quit within the past year and current smokers who smoked for at least 2 years (5).

The prevalence of recent successful cessation (quitting smoking for 6 months or longer within the past year) was examined overall and by sex, race/ethnicity, age, education, occupation, annual household income, metropolitan status, US region, disability/limitation status, current e-cigarette use, current use of other noncigarette tobacco products, and past-year menthol cigarette smoking. Cessation correlates included receipt of advice to quit from a medical doctor, smoke-free home rules, comprehensive smoke-free workplace policy, and use of a cessation treatment/method in the past 12 months. Data were weighted to account for the complex survey design and to yield nationally representative estimates with 95% confidence intervals. Statistical analyses were performed by using SAS-callable SUDAAN version 11.0.1 (Research Triangle Institute). Chi-squared tests were used to determine significant (*P* < .05) differences.

## Results

In 2018, 7.1% of adult smokers reported recent successful cessation (Table 1). By demographic characteristics, no significant differences in the prevalence of recent successful cessation were observed by sex, race/ethnicity, annual household income, metropolitan status, or US region. By age, the prevalence of recent successful cessation of at least 6 months generally decreased as age increased, falling from 13.7% among adults aged 18 to 24 years to 5.0% among adults aged 45 to 64 years. Moreover, prevalence generally increased with greater educational attainment, ranging from 4.4% among adults with less than a high school education to 8.7% among respondents who had at least some college education. By occupation, recent successful cessation of at least 6 months ranged from 4.5% (construction workers) to 8.7% (both white-collar workers and workers in the service industry). Adults without a disability had a significantly higher prevalence of recent successful cessation of at least 6 months (7.5%) than adults with a disability (5.3%). Results also varied by respondents’ use of noncigarette tobacco products. Specifically, current exclusive e-cigarette users had a higher prevalence of recent successful cigarette smoking cessation (15.1%) than adults who currently used other noncigarette tobacco products (3.3%) and adults who did not currently use any other noncigarette tobacco products (6.6%). Prevalence did not differ significantly between adults who usually smoked menthol cigarettes in the past year and adults who did not.

**Table 1:** Prevalence of Recent Successful Cessation^a^ Among Adult Cigarette Smokers, Overall and by Sociodemographic Characteristics, Tobacco Use Supplement to the Current Population Survey, United States, 2018

Demographic Characteristic	Recent Successful Smoking Cessation, % (95% CI)	*P* Value^b^
**Overall**	7.1 (6.4–7.9)	NA
**Sex**		
Male	6.8 (5.8–7.9)	.47
Female	7.4 (6.3–8.8)	
**Age, y**		
18–24	13.7 (9.9–18.8)	<.001
25–44	8.2 (7.0–9.6)	
45–64	5.0 (4.1–6.0)	
≥65	5.8 (4.3–7.8)	
**Race/ethnicity**		
Non-Hispanic White	7.1 (6.3–8.1)	.17
Non-Hispanic Black	4.7 (3.2–7.0)	
Non-Hispanic American Indian/Alaska Native	—c	
Non-Hispanic Asian/Pacific Islander	—c	
Hispanic	8.2 (5.6–11.7)	
Other	—d	
**Education**		
Less than high school	4.4 (3.0–6.3)	<.001
High school graduate/GED	6.2 (4.9–7.7)	
Some college	8.7 (7.4–10.3)	
College graduate	8.5 (6.5–11.1)	
**Annual household income, $**		
<25,000	5.9 (4.6–7.6)	.25
25,000–49,999	7.2 (5.8–9.0)	
50,000–99,999	7.5 (6.2–9.1)	
≥100,000	8.3 (6.4–10.7)	
**Occupation**		
White collar^e^	8.7 (7.3–10.4)	.03
Service^f^	8.7 (6.3–11.9)	
Blue collar^g^	6.6 (4.7–9.2)	
Construction^h^	4.5 (2.4–8.0)	
**Metropolitan status^i^**		
Metropolitan	7.4 (6.6–8.3)	.098
Nonmetropolitan	5.7 (4.5–7.2)	
**US region^j^**		
Northeast	6.1 (4.5–8.2)	.52
Midwest	7.0 (5.6–8.7)	
South	7.0 (5.9–8.3)	
West	8.2 (6.4–10.5)	
**Disability/limitation^k^**		
Yes	5.3 (4.1–6.9)	.006
No	7.5 (6.7–8.4)	
**Noncigarette tobacco product use^l^**		
Current e-cigarette use without use of other noncigarette tobacco products	15.1 (11.2–20.1)	<.001
Current use of noncigarette tobacco products other than e-cigarettes	3.3 (1.8–6.0)	
Current use of e-cigarettes together with other noncigarette tobacco products	—c	
No current noncigarette tobacco product use (including e-cigarettes)	6.6 (5.8–7.4)	
**Past-year menthol cigarette smoking^m^**		
Yes	8.3 (6.8–10.0)	.07
No	6.5 (5.6–7.5)	

Abbreviations: GED, general education diploma; NA, not applicable.

a Recent successful smoking cessation was defined as quitting smoking within the past year for ≥6 months among current cigarette smokers who smoked for ≥2 years and among former smokers who quit during the past year.

b Determined by using χ^2^ test; significance set at *P* < .05. Data were weighted to account for the complex survey design.

c Estimate not presented because relative standard error is >30%.

d Insufficient data.

e White-collar occupations were defined as management; business and financial operations; computer and mathematical science; architecture and engineering; life, physical, and social science; community and social service; legal; education, training, and library; arts, design, entertainment, sports, and media; health care practitioner and technical; sales; and office and administrative support occupations.

f Service occupations were defined as health care support; protective service; food preparation and serving related; building and grounds cleaning and maintenance; and personal care and service occupations.

g Blue-collar occupations were defined as installation, maintenance, and repair; production; and transportation and material moving occupations.

h Construction occupations were defined as construction and extraction occupations.

i Metropolitan was defined as metropolitan statistical area having at least 1 urbanized area of 50,000 or more inhabitants.

j Northeast: Connecticut, Maine, Massachusetts, New Hampshire, New Jersey, New York, Pennsylvania, Rhode Island, and Vermont; Midwest: Illinois, Indiana, Iowa, Kansas, Michigan, Minnesota, Missouri, Nebraska, North Dakota, Ohio, South Dakota, and Wisconsin; South: Alabama, Arkansas, Delaware, District of Columbia, Florida, Georgia, Kentucky, Louisiana, Maryland, Mississippi, North Carolina, Oklahoma, South Carolina, Tennessee, Texas, Virginia, and West Virginia; West: Alaska, Arizona, California, Colorado, Hawaii, Idaho, Montana, Nevada, New Mexico, Oregon, Utah, Washington, and Wyoming.

k Respondents who reported deafness or difficulty hearing; blindness or difficulty seeing; serious difficulty concentrating, remembering, or making decisions because of a physical, mental, or emotional condition; serious difficulty walking or climbing stairs; difficulty dressing or bathing; or difficulty doing errands alone such as visiting a doctor’s office or shopping because of a physical, mental, or emotional condition.

l Current e-cigarette use was defined as using e-cigarettes every day or some days; current other tobacco use was defined as using regular cigars, cigarillos, little filtered cigars, regular pipe filled with tobacco, water pipe or hookah pipe filled with tobacco, smokeless tobacco, or dissolvable tobacco every day or some days; and no current tobacco product use was defined as not using e-cigarettes, regular cigars, cigarillos, little filtered cigars, regular pipe filled with tobacco, water pipe or hookah pipe filled with tobacco, smokeless tobacco, and dissolvable tobacco every day or some days.

m Past-year menthol cigarette smoking was defined as usually smoking menthol cigarettes during the 12 months before quitting smoking.

By cessation correlates, recent successful cessation was higher among adult smokers who were advised to quit smoking by a medical doctor in the past year (4.9%) than among adults who were not (3.2%) and among adults who reported having smoke-free home rules (9.8%) than among adults who did not (2.4%) (Table 2). Recent successful cessation did not differ significantly by the presence of a comprehensive smoke-free workplace policy or by cessation methods used in the past year, which ranged from 10.4% for adults who used nicotine replacement therapy alone to 15.1% for adults who did not use any cessation method.

**Table 2:** Prevalence of Recent Successful Cessation^a^ Among Adult Cigarette Smokers, by Correlates, Tobacco Use Supplement to the Current Population Survey, United States, 2018

Correlate	Recent Successful Smoking Cessation, % (95% CI)	*P* Value^b^
**Advised to quit by medical doctor in past year**		
Yes	4.9 (4.0–6.0)	.04
No	3.2 (2.1–5.0)	
**Smoke-free rule in home**		
Yes	9.8 (8.8–10.9)	<.001
No	2.4 (1.7–3.3)	
**Comprehensive smoke-free workplace policy^c^**		
Yes	8.2 (6.7–10.0)	.33
No	10.2 (7.2–14.3)	
**Cessation method used in past year**		
NRT only^d^	10.4 (7.0–15.1)	.10
Non-nicotine pharmacotherapy only^e^	13.6 (7.8–22.6)	
Counseling only^f^	––g	
NRT/pharmacotherapy and counseling only	––g	
Switch to other tobacco products only, excluding e-cigarettes^h^	––g	
Switch to e-cigarettes only^i^	12.7 (9.1–17.6)	
None of the above	15.1 (13.1–17.4)	

Abbreviation: NRT, nicotine replacement therapy.

a Recent successful smoking cessation was defined as quitting smoking within the past year for ≥6 months among current cigarette smokers who smoked for ≥2 years and among former smokers who quit during the past year.

b Determined by using χ^2^ test; significance set at *P* < .05. Data were weighted to account for the complex survey design.

c Comprehensive smoke-free workplace policy was defined as a policy under which smoking was not allowed in any indoor public or common area or any indoor work areas.

d Includes respondents who indicated only using the following when they tried to quit smoking in the past year: a nicotine patch, gum, lozenge, nasal spray, or inhaler.

e Includes respondents who indicated using only the following when they tried to quit smoking in the past year: a prescription pill called Chantix, Varenicline, Zyban, Bupropion, or Wellbutrin.

f Includes respondents who indicated using only the following when they tried to quit smoking cigarettes in the past year: a telephone help line or quit line; one-on-one in-person counseling by a health professional; a stop-smoking clinic, class, or support group; an internet or web-based program or tool, including smartphone apps and text-messaging programs.

g Estimate not presented because relative standard error is >30%.

h Includes respondents who indicated that they tried to quit smoking cigarettes by switching to smokeless tobacco such as chewing tobacco, snuff, or snus; regular cigars, cigarillos, or little filtered cigars; or any pipes filled with tobacco. Those who reported switching to e-cigarettes were excluded.

i Includes respondents who indicated that they tried to quit smoking cigarettes by switching to e-cigarettes but not any other tobacco products.

## Discussion

We found that in 2018, 7.1% of US adult smokers reported quitting smoking for 6 months or longer. This finding aligns with an estimate of 7.5% based on 2018 National Health Interview Survey data (6). Furthermore, a subset of adults who successfully quit smoking reported current use of noncigarette tobacco products and thus continued to be exposed to the harmful effects of tobacco.

Prior data demonstrate that smokers use various evidence-based and nonevidence-based methods when trying to quit (7). In our study, no single cessation method or combination of methods assessed was significantly associated with recent successful cessation; however, small cell sizes limited the ability to obtain several estimates. Currently, 7 medications approved by the Food and Drug Administration (FDA) and 3 types of counseling are scientifically proven to be safe and effective in helping adult smokers quit (2,8,9).

Our findings indicate that 15% of smokers who were current exclusive users of e-cigarettes reported recent successful smoking cessation. The role of e-cigarettes in helping smokers transition completely away from cigarette smoking warrants further research; the US Surgeon General’s report concluded evidence is inadequate to conclude that e-cigarettes, in general, increase smoking cessation (2), and the FDA has not approved e-cigarettes as safe and effective smoking cessation aids (2,10).

This study has limitations. First, smoking status and cessation behaviors were based on self-report. Second, these data are cross-sectional and cannot be used to assess temporality or causality; therefore, the association between certain indicators, including current e-cigarette use and recent successful cessation, should be interpreted with caution (11). Third, the study did not assess other factors (eg, health insurance status) that could contribute to differences in recent successful cessation among adult smokers.

In conclusion, in 2018, about 1 in 14 US adult smokers reported recent successful smoking cessation. Some groups had less success, including certain demographic groups, and some groups had greater success, including exclusive e-cigarette users, people with smoke-free home rules, and people who received advice to quit from a medical doctor. To help more smokers quit, public health practitioners can ensure that evidence-based tobacco control interventions, including barrier-free access to evidence-based cessation treatments, are reaching populations that face greater barriers to successfully quitting smoking (1,2). Coordinated local, state, and national efforts can accelerate progress toward increasing smoking cessation and reducing tobacco-related disease and death (1,2).
